# Hypoglycemia Reporting in GLP‐1RA Trials for Type 1 Diabetes: A Critical Gap That Needs To Be Addressed

**DOI:** 10.1002/edm2.70256

**Published:** 2026-06-05

**Authors:** Saad Ishtiaq, Zain Mehboob Malik, Abdur Rahim Ahmad

**Affiliations:** ^1^ Khyber Medical University Peshawar Pakistan; ^2^ Abbottabad International Medical Institute Abbottabad Pakistan

Dear Editor,

1

We read with great interest the systematic review and meta‐analysis by Chen et al., which thoroughly assessed the impact of glucagon‐like peptide‐1 receptor agonists (GLP‐1RAs) on cardiometabolic risk factors when used as add‐on therapy in type 1 diabetes mellitus (T1DM) [1]. The authors highlight statistically significant improvements in several key areas, including systolic and diastolic blood pressure, LDL cholesterol, C‐reactive protein (CRP), HbA1c, body weight, and BMI, based on their analysis of 21 randomized controlled trials (RCTs) involving 3417 patients. These findings carry important clinical implications, and we commend the authors for the wide range of outcomes they examined and their adherence to PRISMA 2020 guidelines.

However, we feel it's important to point out a clinically significant omission: the lack of any systematic analysis of the incidence and severity of hypoglycemia. In T1DM, where patients rely entirely on external insulin, this isn't just a secondary concern but a primary safety issue.

The reason for this concern is well‐known from a pharmacological perspective. GLP‐1RAs reduce post‐meal glucagon secretion, slow down gastric emptying, and boost glucose‐dependent insulin release. While these mechanisms can be beneficial on their own, they might intensify the risk of hypoglycemia when combined with insulin therapy. For instance, the ADJUNCT ONE trial revealed significantly higher rates of symptomatic hypoglycemia with higher liraglutide doses in T1DM patients—16.5 events per patient per year at the 1.8 mg dose, compared with 12.3 per patient per year with placebo. This was accompanied by increased rates of hyperglycemia with ketosis, leading the trial investigators to conclude that the clinical usefulness of liraglutide in this population is limited [2]. Similarly, the ADJUNCT TWO trial confirmed higher rates of symptomatic hypoglycemia with liraglutide 1.2 mg compared to placebo (21.3 vs. 16.6 events per patient per year), reinforcing the need for careful insulin dose adjustments when using these agents as supplementary treatments [2]. Real‐world data from the FDA Adverse Event Reporting System (FAERS) has also identified hypoglycemia signals across various GLP‐1RA agents [3]. Although a previous meta‐analysis didn't find a statistically significant increase in the frequency of severe hypoglycemia with GLP‐1RA use in T1DM [4], the differences between the included trials—particularly in how severe hypoglycemia was reported across the 13 studies—mean that a pooled finding of no significant increase shouldn't be taken as proof of no risk [4].

This omission is particularly important because the same add‐on therapy that produces the favourable HbA1c reduction (WMD: −0.21%) reported by Chen et al. could also lower the threshold for hypoglycemia, especially if insulin doses aren't adjusted downwards accordingly. Without data on hypoglycemia rates alongside data on glycemic efficacy, clinicians are left with an incomplete picture of the risks and benefits, which could lead to unsafe clinical decisions.

We respectfully suggest that future versions of this meta‐analysis, or a separate systematic review, should include hypoglycemia as a pre‐specified primary safety outcome, broken down by: (i) severity—mild, moderate, and severe; (ii) timing—nocturnal versus daytime; and (iii) GLP‐1RA agent and dose. Using the International Hypoglycemia Study Group (IHSG) standardized three‐level classification—level 1 (glucose ≤ 3.9 mmol/L), level 2 (glucose < 3.0 mmol/L), and level 3 (severe cognitive impairment requiring third‐party assistance)—across the included trials would improve comparability and has already been endorsed by the American Diabetes Association, the European Association for the Study of Diabetes, and the European Medicines Agency [5].

Addressing this gap is crucial if GLP‐1RAs are to be safely and confidently recommended as an add‐on therapy for people with T1DM.

## Author Contributions

**Saad Ishtiaq:** conceptualization, writing – original draft. **Zain Mehboob Malik:** validation, software. **Abdur Rahim Ahmad:** formal analysis, software.

## Funding

The authors have nothing to report.

## Ethics Statement

The authors have nothing to report.

## Consent

The authors have nothing to report.

## Conflicts of Interest

The authors declare no conflicts of interest.

## Data Availability

No new data was generated in this letter.
